# Recent Advances in the Surgical Treatment of Differentiated Thyroid Cancer: A Comprehensive Review

**DOI:** 10.1155/2013/425136

**Published:** 2013-01-08

**Authors:** Raghunandan Venkat, Marlon A. Guerrero

**Affiliations:** ^1^Division of Surgical Oncology, Department of Surgery, University of Arizona, P.O. Box 245131, Tucson, AZ 85724-5131, USA; ^2^Division of Surgical Oncology, Arizona Cancer Center, Tucson, AZ 85719, USA

## Abstract

Differentiated thyroid cancers have become one of the fastest growing malignancies in the world. While surgery has remained the cornerstone of management of these tumors, the surgical approach has seen numerous innovations over the past few decades. The use of video-assistance and robotics has revolutionized thyroid surgery. This paper provides a comprehensive evaluation of the different approaches to thyroid surgery, the utility of prophylactic and therapeutic lymph node dissection, and evidence-based guidelines in the treatment of differentiated thyroid cancers. Minimally invasive video-ssisted thyroidectomy is both safe and effective in the hands of the trained surgeon and, in selected patient populations, has comparative perioperative morbidity and better cosmesis as compared to conventional open thyroidectomy. It is universally accepted that therapeutic central lymph node dissection should be performed when metastatic lymph nodes are identified on physical exam, ultrasound, or intraoperatively. In the absence of overt nodal metastasis, the role of elective prophylactic central lymph node dissection remains a matter of debate and prospective, randomized studies are warranted to evaluate the utility of this procedure.

## 1. Introduction

In the past 20 years, differentiated thyroid cancer (DTC) has become the fastest growing malignancy in the world [1]. The rise in the incidence of thyroid cancer (3.01%) is the highest in the United States with over 48,000 cases annually [2]. DTCs arise from follicular cells and include papillary (PTC), follicular (FTC), and Hürthle cell carcinoma (HCC). Surgical resection has remained the gold standard for the treatment for DTC. 

Over the last two decades, there have been numerous changes in the surgical approach to thyroidectomy. Many of these include instrument innovations, such as the LigaSure, harmonic scalpel, and intraoperative nerve monitoring; while other major advances include minimally invasive surgical procedures with video assistance and robotic surgery. These changes have led to a paradigm shift in the surgical treatment of DTC.

The aim of this paper is to provide a comprehensive evaluation of the various approaches to the surgical treatment of DTC (conventional open, minimally invasive video-assisted, and endoscopic and robotic thyroidectomies) in terms of operative technique, clinical outcomes, and oncologic feasibility. We further evaluate the risks and benefits of prophylactic central lymph node dissection (CND). 

## 2. Methods

A review of the literature was performed using Medline and Pubmed databases to identify all studies published up to October 2012 involving thyroidectomy for thyroid cancer. The MeSH search terms used were “thyroid neoplasms,” “thyroidectomy,” “endoscopy,” and “video-assisted surgery.” The above terms and their combinations were also searched as text words, as were the terms “differentiated thyroid cancer,” their subtypes (papillary, follicular, and Hürthle cell) and “robot assisted.” We excluded studies involving cancers of parafollicular origin or advanced differentiated thyroid cancer. 

## 3. Results

Our search strategy yielded 443 studies related to the surgical treatment of DTC and we included 45 studies that discussed different approaches to the surgical treatment of DTC (conventional open, minimally invasive video-assisted, endoscopic, and robotic thyroidectomies) in terms of operative technique, clinical outcomes and oncologic feasibility as well as the utility of prophylactic and therapeutic lymph node dissection.

### 3.1. Risk Stratification

 Various studies have evaluated risk factors and developed risk stratification systems for thyroid cancer [3–5]. The prognostic factors include age at diagnosis, tumor size, grade of tumor, gender, extrathyroidal extension, lymph node involvement, completeness of resection, positive margins, multicentricity, and presence of distant metastasis. Tuttle et al. [5] classified risk of death from thyroid cancer into four categories (Table 1): very low risk, low risk, intermediate risk, and high risk. Low risk features include young age at diagnosis, classical histology of PTC confined to the thyroid gland with no evidence of vascular invasion, smaller tumors (≤4 cm), complete resection, no evidence of distant metastasis, or cervical lymph node involvement. High risk features include age at diagnosis >45 years, larger tumors (>4 cm) or worrisome histology (PTC subtypes such as tall cell, columnar or insular variants, and poorly differentiated thyroid cancers), incomplete resection, vascular invasion, cervical lymph node involvement, and distant metastasis.

 Histologically some variants of PTC have been reported to behave more aggressively. For instance the tall cell variant of PTC, which was first described by Hawk and Hazard [20] and comprises 5–10% of all cases, is more likely to be associated with high risk features such as larger size, extrathyroidal extension and distant metastasis [21]. They also have a higher incidence of progression to anaplastic carcinoma and have a higher recurrence rate and mortality, thus warranting aggressive treatment approaches [21, 22].

### 3.2. Surgical Approaches

 Surgery remains the mainstay treatment for DTC. Total/near-total thyroidectomy and thyroid lobectomy, with or without isthmusectomy, are the two most accepted options. Total thyroidectomy is the removal of the entire thyroid gland, while preserving the parathyroid glands and the recurrent and superior laryngeal nerves. In near total thyroidectomy, which is considered equal to total thyroidectomy, a small amount of posterior thyroid capsule remains. In thyroid lobectomy, the contralateral gland is not removed. Total/near-total thyroidectomy is considered the procedure of choice for most DTCs [23]. Although some studies have shown comparable long-term results between thyroid lobectomy and total thyroidectomy in low-risk and select intermediate-risk patients [24], Bilimoria et al. [6] using National Cancer Database reported that lobectomy alone resulted in a higher risk of recurrence (hazard ratio: 1.15, *P* = 0.04) and death (hazard ratio: 1.31, *P* = 0.009) in tumors >1 cm compared to total/near-total thyroidectomy. Several studies have supported this recommendation (Tables 2 and 3) [6–9]. Moreover, studies have shown a 35–60% rate of occult cancer and a 6–10% rate of recurrence in the contralateral lobe. Furthermore, the removal of the entire thyroid gland facilitates the use of radioactive iodine for adjuvant therapy, measurement of serum thyroglobulin for disease surveillance, and neck ultrasonography to identify residual and/or recurrent disease. For small tumors, <1 cm confined to one thyroid lobe, with no contralateral nodules, thyroid lobectomy is an acceptable alternative. Thyroid lobectomy is a more limited procedure that avoids placing the contralateral recurrent laryngeal nerve and the parathyroid glands at risk for injury [23]. Some studies have shown greater recurrence rates with thyroid lobectomy [6], however, long-term survival does not seem to be affected [24].

 Conventional open thyroid surgery, described initially by Dr. Emil Kocher [25], has been the standard surgical technique for almost a century. This initially involved a 8–10 cm transverse midline neck incision and, over the years, greatly reduced to standard a 3–6 cm incision [26]. Although this method is quick, provides excellent exposure, and leaves a scar hidden in the skin crease, the risk of scar hypertrophy and search for better cosmetic results have led to the development of minimally invasive techniques, such as video-assistance, endoscopy, and robotic surgery. 

#### 3.2.1. Endoscopic Thyroid Surgery

 Endoscopic thyroid surgery was first described in 1997 by Huscher et al. [27] This technique, popularly known as minimally invasive video assisted thyroidectomy (MIVAT) is the most widely accepted endoscopic technique. Developed by Miccoli et al. [28] the video-assisted techniques are divided into three steps: the access to the thyroid bed and the creation of the working space through the minimal skin incision(s); the dissection of the thyroid lobe(s) after the identification of the recurrent laryngeal nerve and the parathyroid glands; and the retrieval of the thyroid lobe(s) and closure of the wounds. These three parts of the operation may last different lengths of time according to the different techniques used. Three main endoscopic approaches have been described for the thyroid gland: the cervical [29], the axillary [30], and the breast/lateral approach [31]. The safety of the video-assisted cervical approach has been established by Miccoli's series of 833 patients [32] and established by numerous reports, which have confirmed a similar complication rate compared to open thyroidectomy, as well as improved cosmesis and faster recovery (Table 4) [10–14].

#### 3.2.2. Robotic Thyroid Surgery

In general, conventional endoscopic surgeries have some limitations in obtaining adequate visualizations and precise, meticulous manipulation of the surgical tissues. These limitations result from the two-dimensional representation and the simplicity of the endoscopic instruments used. The da Vinci S surgical robot system (Intuitive Surgical, Sunnyvale, CA, USA) was developed to address these limitations of conventional endoscopic surgery This procedure was initially described by Kang et al. [33] and avoids the use of a neck incision altogether. However, it has been shown that operative times are longer, and there is a significant learning curve for the procedure (Table 4) [34]. Additionally, it is subject to increased cost for the robot and potentially longer operative times, and some would argue that the procedure is more invasive due to the dissection needed to approach from the axilla across the chest to reach the thyroid.

### 3.3. Lymph Node Dissection: Prophylactic or Therapeutic?

 Patients with DTC commonly have lymph node involvement. While up to 20–90% patients with PTC may have lymph node metastasis detected during the initial surgery, the rate of lymph node involvement is substantially lower (2%) with follicular thyroid cancer (FTC) [35–37]. Although lymph node status is not a part of several staging systems, such as the AGES [38] and the AMES [4], it is used to stratify prognosis in patients older than 45 years with DTC according to the AJCC [39]. 

 The central neck or level VI lymph node compartment is anatomically bounded by the hyoid bone superiorly, the innominate artery inferiorly, and the carotid sheath laterally [40]. Since the recurrent laryngeal nerves and the parathyroid glands are situated in this compartment, careful surgical dissection is required to preserve function of these structures. 

It is universally accepted that a therapeutic CND should be performed; metastatic lymph nodes are identified on physical exam, ultrasound, or intraoperatively [23]. Therapeutic lymph node dissection decreases the incidence of locoregional recurrence (by up to 2–7%), prevent local progression into adjacent structures, and improve survival (by up to 3–9%) [36, 41, 42]. 

 In the absence of overt nodal metastasis, the role of elective prophylactic central lymph node dissection remains a matter of debate [41, 43]. Unanticipated microscopic metastases are identified in 38–45% patients undergoing prophylactic CND [19, 44]. However, preoperative radiologic evaluation of the central compartment is limited by the overlying thyroid gland. Furthermore, intraoperative inspection is highly inaccurate in identifying lymph node involvement [45, 46]. 

 The American Thyroid Association (ATA) guidelines recommend performing prophylactic CND in patients with PTC and locally advanced primary tumors (T3 and T4) [23]. This recommendation is based on evidence from retrospective studies [47, 48]. Scheumann et al. had reported decreased recurrence (*P* < 0.00001) and improved survival (*P* = 0.005) in 342 patients with T1–T3 disease who had total thyroidectomy with CND as compared to total thyroidectomy alone [47]. The ATA guidelines recommend thyroidectomy without prophylactic CND for small (T1 and T2), noninvasive and clinically node negative PTC and most FTC. Patients meeting these criteria have a lower risk of lymph node metastasis and are less likely to benefit from additional surgery. The European Thyroid Cancer Taskforce also recommends prophylactic CND only in patients with preoperatively suspected and/or intraoperatively proven lymph node metastasis [47]. A recent systematic review and meta-analysis of 16 retrospective studies by Shan et al. [49] reported no difference in recurrence rates, rate of recurrent laryngeal nerve injuries (temporary or permanent), or permanent hypocalcemia between total thyroidectomy with CND as compared to total thyroidectomy alone. Temporary hypocalcemia was seen to be more common in the CND group (Table 5) [15–19]. In the light of these conflicting reports, the role of prophylactic CND is still a topic of considerable debate and larger prospective trials are needed to evaluate the benefit of prophylactic CND in DTC.

 Thyroid cancer can also metastasize to the lateral compartment of lymph nodes comprising of levels II–V. Suspicious lymph nodes in the lateral compartment [40] should be biopsied by FNA, and if positive, a modified radical lymphadenectomy should be performed. Studies have shown that cytoreductive surgery is associated with decreased recurrence and improved survival [45, 50, 51], however an en block resection can be associated with significant morbidity, including long-term motor dysfunction. Thus in patients with minimal disease, a limited lymphadenectomy is desirable. Nodal metastasis is most commonly found in level III, followed by levels IV and II, with level V being least common [52, 53]. Therefore, it is well accepted to perform a targeted compartmental lymph node dissection, aided by preoperative assessment, while “berry picking” or isolated lymphadenectomy is discouraged.

 Patients at risk for developing aggressive disease can be identified using molecular testing for gene alterations present in PTC, such as BRAF and RAS as well as RET-PTC and PAX8-PPARG rearrangements [54–56]. Such patients could derive increased benefit from prophylactic CND [54, 56]. Although the utility of molecular testing has not yet been prospectively evaluated in randomized trails, testing for BRAF, which is the best studied thyroid oncogene, is routinely performed in some institutions to guide decision making in patients with PTC [57].

## 4. Conclusion

DTC has become an increasingly common malignancy. It is well accepted that surgery remains the mainstay of treatment of this disease and there have been tremendous advances in the approach to surgery over the last two decades. MIVAT and robotic thyroidectomy are seen to be safe and effective approaches in the hands of the trained surgeon and in selected patient populations. However, there is still considerable debate regarding the role of prophylactic lymph node dissection in the absence of preoperative or intraoperative signs of nodal metastasis. Randomized and prospective studies are warranted to shed more light on the indications for this procedure.

## Figures and Tables

**Table 1:** Risk of death from thyroid cancer [5].

	Very low risk	Low risk	Intermediate risk	High risk
Age at diagnosis	<45 years	<45 years	Young patients (<45 years) Classic PTC >4 cm Vascular invasion Extrathyroidal extension Worrisome histology of any size^‡^	>45 years

Primary tumor size	<1 cm	1–4 cm	Older patients (>45 years) Classic PTC <4 cm Extrathyroidal extension Worrisome histology <1-2 cm confined to the thyroid^‡^	>4 cm classic PTC

Histology	Classic PTC, confined to the thyroid gland*	Classic PTC, confined to the thyroid gland*	Histology in conjunction with age as above	Worrisome histology >1-2 cm^‡^

Completeness of resection	Complete resection	Complete resection	Complete resection	Incomplete tumor resection

Lymph node involvement	None apparent	Present or absent^†^	Present or absent^†^	Present or absent^†^

Distant metastasis	None apparent	None apparent	None apparent	Present

Only those patients meeting all criteria within the respective column would be classified as very low risk or low risk. Older patients with either incomplete tumor resection or presence of distant metastasis are considered high risk irrespective of tumor size and specific histology. Patients with a combination of risk factors (age, histology, and tumor size) crossing over between columns are classified as intermediate-risk patients. PTC: papillary thyroid cancer.

*Confined to the thyroid gland with no evidence of vascular invasion or extrathyroidal extension.

^†^Cervical lymph node metastases in older patients, but probably not in younger patients, may confer an increased risk of death from disease.

^‡^Worrisome histologies include histologic subtypes of papillary thyroid cancer such as tall cell variant, columnar variant, insular variant, and poorly differentiated thyroid cancers.

**Table 2:** Comparison of outcomes of lobectomy and total thyroidectomy.

Study	Recurrence (%)	Survival (%)
	10-year^+^	10-year^+^
Bilimoria et al. [6] *N* = 52,173	TL: 9.8	TL: 97.1
	TT: 7.7	TT: 98.4

	10-year^+^	10-year
Mazzaferri and Young [7] *N* = 576	TL: 19.2	TL: 98.5
	TT: 10.9	TT: 99.4

	30-year^+^	30-year
Hay et al. [8] (*N* = 1685)	TL: 22.2	TL: 97.6
	TT: 8.3	TT: 97.4

TT: total thyroidectomy, TL: thyroid lobectomy.

^+^Statistically significant (*P* ≤ 0.05).

**Table 3:** Comparison of outcomes of by tumor size (cm).

Study	Recurrence (%)	Survival (%)
	10-year^+^	10-year
Mazzaferri and Young [7] *N* = 576	<1.5 : 4.8	<1.5 : 100.0
	≥1.5 : 12.7	≥1.5 : 97.9

	30-year^+^	30-year
Mazzaferri and Jhiang [9] *N* = 1355	<1.5 : 8	<1.5 : 100
	1.5–4.4 : 31	1.5–4.4 : 94
	≥4.5 : 36	≥4.5 : 86

	10-year^+^	10-year
	<1 : 4.6	<1 : 98.0
	1–1.9 : 7.1	1–1.9 : 98.4
Bilimoria et al. [6] *N* = 52,173	2–2.9 : 8.6	2–2.9 : 98.5
	3–3.9 : 11.6	3–3.9 : 95.5
	4–8 : 17.2	4–8^+^ : 90.5
	>8 : 24.8	>8^+^ : 81.3

^+^Statistically significant (*P* ≤ 0.05).

**Table 4:** Comparison of outcomes of open, endoscopic and robot assisted thyroidectomy.

Study	Surgery	Size (cm)	CND (%)	Operative time (min)	Complications (%)				
					Transient hypocalcemia	Permanent hypocalcemia	Transient RLN palsy	Permanent RLN palsy	Hematoma
Jong et al. [10]	O (*n* = 224) E (*n* = 275)	O: 0.60 ± 0.22 E: 0.56 ± 0.19	Prophylactic ipsilateral in all patients	O: 105.5 ± 41.6 E: 138.5 ± 49.0	O: 17.1 E: 31.0	O: 1.7 E: 0.0	O: 2.2 E: 5.5	O: 0.4 E: 0.4	O: 0.9 E: 1.5

Di et al. [11]	O (*n* = 37) E (*n* = 31)	O: 0.73 ± 0.14 E: 0.76 ± 0.15	All patients underwent CND	O: 105.4 ± 37.0 E: 143.9 ± 19.2	O: 2.7 E: 9.7	O: 0.0 E: 0.0	O: 5.4 E: 9.7	O: 0.0 E: 0.0	O: 0.0 E: 0.0

Kim et al. [12]	O (*n* = 138) E (*n* = 95) R (*n* = 69)	O: 0.7 ± 0.2 E: 0.6 ± 0.2 R: 0.6 ± 0.2	Prophylactic ipsilateral in all patients	O: 81 ± 16 E: 136 ± 31 R: 196 ± 45	O: 27.5 E: 25.3 R: 33.3	O: 0.0 E: 2.1 R: 0.0	O: 0.7 E: 2.1 R: 1.4	O: 0.0 E: 2.1 R: 0.0	O: 0.0 E: 1.1 R: 0.0

Lee et al. [13]	E (*n* = 96) R (*n* = 163)	E: 0.84 ± 0.41 R: 0.87± 0.64	E: 54.2 R: 91.4	E: 142.7 ± 52.1 R: 110.1 ± 50.7	E: 0.0 R: 12.5	E: 0.0 R: 0.0	E: 3.1 R: 1.8	E: 1.0 R: 0.6	E: 3.1 R: 0.6

Chung et al. [14]	O (*n* = 198) E (*n* = 103)	≤0.5 cm (%) O: 52.0 E: 56.3 0.5–1 cm (%) O: 48.0 E: 43.7	O: 8.1 E: 1.0	O: 111.4 E: 165.1 (S.D. not reported)	O: 17.7 E: 25.2	O: 4.5 E: 1.0	O: 2.5 E: 25.2	O: 0.5 E: 0.0	O: 0.0 E: 1.0

O: open thyroidectomy, E: endoscopic thyroidectomy, R: robot assisted thyroidectomy, CND: central lymph node dissection, S.D.: standard deviation, continuous variable reported as mean ± S.D., categorical variables reported as percentages (%).

**Table 5:** Comparison of outcomes of total thyroidectomy with or without central lymph node dissection.

Study	RLN Injury (%)		Hypocalcemia (%)		Recurrence (%)
	Temporary	Permanent	Temporary	Permanent	
Shen et al. [15]	TT: 3.7	TT: 1.0	TT: 11.0	TT: 0	TT: 21.8
(*N* = 301)	TT + CND: 1.8	TT + CND: 1.8	TT + CND: 38.2	TT + CND: 0	TT + CND: 5.7

Moo et al. [16]	TT: 0	TT: 0	TT: 5.6	TT: 5.5	TT: 16.7
(*N* = 81)	TT + CND: 4.4	TT + CND: 0	TT + CND: 31.1	TT + CND: 0	TT + CND: 4.4

Rosenbaum McHenry [17]	TT: 2.3	TT: 1.1	TT: 57.9	TT: 0	TT: 4.5
(*N* = 110)	TT + CND: 9.1	TT + CND: 0	TT + CND: 86.4	TT + CND: 4.5	TT + CND: 2.3

Perrino et al. [18]	TT: 3.1	TT: 2.5	TT: 6.9	TT: 3.8	TT: 13.8
(*N* = 251)	TT + CND: 2.2	TT + CND: 1.1	TT + CND: 8.7	TT + CND: 1.1	TT + CND: 5.4

Sywak et al. [19]	TT: 1.0	TT: 1.0	TT: 8.2	TT: 0.5	TT: 5.6
(*N* = 447)	TT + CND: 1.8	TT + CND: 0	TT + CND: 17.8	TT + CND: 1.8	TT + CND: 3.6

TT: total thyroidectomy alone, TT + CND: total thyroidectomy with central lymph node dissection.
